# From hepatoprotection models to new therapeutic modalities for treating liver diseases: a personal perspective

**DOI:** 10.12688/f1000research.8609.2

**Published:** 2016-07-19

**Authors:** Swetha Rudraiah, José E. Manautou

**Affiliations:** 1 Toxicology Program, Department of Pharmaceutical Sciences, School of Pharmacy, University of Connecticut, Storrs, CT, USA

**Keywords:** hepatoprotection, liver diseases, acetaminophen, acute liver failure, APAP toxicity

## Abstract

A variety of rodent models of hepatoprotection have been developed in which tolerance to acetaminophen-induced hepatotoxicity occurs. Autoprotection/heteroprotection is a phenomenon where prior exposure to a mildly toxic dose of toxicant confers protection against a subsequently administered higher dose of the same toxicant (as in the case of autoprotection) or to a different toxicant (referred to as heteroprotection). Multiple mechanisms regulate this adaptive response, including hepatocellular proliferation, proteostasis, enhanced expression of cytoprotective genes, and altered tissue immune response. In this review, we will discuss recent findings that highlight the complexity of these adaptive mechanisms and we also outline the usefulness of these findings to devise therapeutic and/or diagnostic tools for acetaminophen-induced liver damage in patients.

## Introduction

Acetaminophen (APAP) continues to be one of the most commonly used analgesic and antipyretic agents in the U.S. and Great Britain. Its therapeutic benefits were first described more than a century ago. However, APAP poisoning and its related hepatotoxicity were first recognized in Great Britain ^1^ and later in the U.S. ^2^. APAP usage and popularity increased after aspirin was implicated in over 80% of Reye’s syndrome cases in children. As APAP usage increased, the incidence of APAP-induced hepatotoxicity increased concurrently. In a retrospective study from 1994 to 1996, APAP-induced hepatotoxicity comprised about 25% of acute liver failure (ALF) cases in the U.S. ^3^. By the early 2000s, nearly 50% of all ALF cases from all etiologies were due to APAP use or misuse ^4^. Alarmingly, these statistics have remained fairly constant over the past decade; APAP use continues to be the most common cause of ALF in western countries with no clear improvement in sight ^4, 5^. The potential for APAP-related morbidity and mortality combined with the drug’s popularity and unrestricted access poses a significant human health problem.

The current treatments for ALF due to APAP include either administration of the glutathione (GSH) precursor N-acetyl cysteine (NAC) or orthotopic liver transplantation (OLT) in those patients where NAC is ineffective. NAC increases the availability of intracellular cysteine, which in turn enhances the GSH formation rate. Thus, replenishment of cellular GSH is the main mechanism through which NAC confers protection against APAP-induced liver injury. There are limitations associated with antidotal NAC treatment, particularly in unintentional APAP-overdose patients who do not report to a doctor until they feel discomfort. NAC is effective when administered during the early stages (up to 24 hours) after APAP overdose, although symptoms frequently do not occur until later ^5^. OLT remains an ultimate choice of treatment in severe cases but is associated with high cost and limited availability of donor livers, also with confounded morbidity and mortality ^6^. These long-standing limitations have prompted extensive research aimed at devising not only new measures to reduce the risk of APAP hepatotoxicity but also new treatment options.

**Figure 1.**
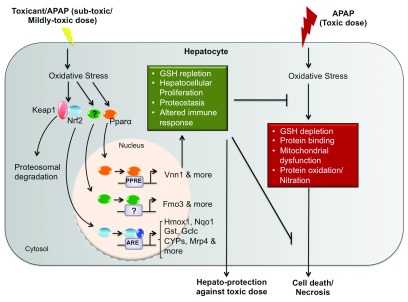
Mechanism of autoprotection/heteroprotection in the hepatocyte. In response to a toxic dose of acetaminophen (APAP), oxidative stress ensues. Resulting molecular events include those depicted here: glutathione (GSH) depletion, protein binding/protein adduct formation, mitochondrial dysfunction, and protein oxidation/nitration leading to cellular death or necrosis. In autoprotection/heteroprotection, a subtoxic or a mildly toxic dose of toxicant (APAP or clofibrate) results in activation of various transcription factors (Nrf2, PPARα, etc.), resulting in the transcription of cytoprotective genes that play a role in adaptive responses. These adaptive responses include hepatocellular proliferation, proteostasis, enhanced expression of cytoprotective genes, and altered tissue immune responses that protect the liver against a challenge toxic dose of APAP. Abbreviations: APAP, acetaminophen; ARE, antioxidant response element; CYP, cytochrome P450; FMO3, flavin-containing monooxygenase 3; Gclc, glutamate-cysteine ligase, catalytic subunit; Gst, glutathione S-transferase; Hmox1, heme oxygenase-1; Keap1, kelch-like ECH-associated protein 1; Mrp, multidrug resistance-associated protein; Nrf2, nuclear factor (erythroid-derived 2)-like 2; Nqo1, NAD(P)H quinone dehydrogenase 1; PPARα, peroxisome proliferator-activated receptor alpha; PPRE, peroxisome proliferator-activated receptor response element; Vnn1, Vanin-1.

Despite decades of considerable effort and steady progress towards understanding the pathophysiological basis of APAP hepatotoxicity and hepatoprotection, an evolution of novel therapeutics has yet to be realized. Significant efforts are underway to investigate the importance of regenerative therapies in ALF patients ^4, 5, 7– 10^. Apte *et al.* show β-catenin activation as one of the mechanisms contributing to spontaneous regeneration following APAP-induced liver injury and its potential use as a regenerative therapy in ALF cases ^9^. The process of adaptation to APAP-induced liver injury is multifactorial and more complex and dynamic than previously thought. The advent of high-throughput gene microarray and proteomic analysis has facilitated the identification of global changes in gene and protein expression. Using these modern tools, novel mechanisms implicated in adaptation to APAP toxicity have been identified in the rodent models of autoprotection/heteroprotection ^11– 17^. An update on the experimental models of hepatoprotection (both autoprotection and heteroprotection) (summarized in Figure 1) and their potential diagnostic/therapeutic usefulness in the prognosis and treatment of APAP-induced hepatotoxicity will be summarized in the current review.

## Autoprotection and heteroprotection

Autoprotection/heteroprotection is an experimental approach employed to modulate APAP hepatotoxicity in rodents. Autoprotection is the resistance to toxicant re-exposure following acute, mild injury with the same toxicant, whereas in heteroprotection, the initial toxicant used is different from the second one. In their pioneering work, Mehendale and colleagues first demonstrated autoprotection in rats in the early 1990s using the hepatotoxicant carbon tetrachloride (CCl _4_) ^18^. Similarly, studies conducted by our group have demonstrated both autoprotection/heteroprotection in mice and that pretreatment of mice with various xenobiotics with different modes of action results in considerable reduction in the severity of APAP toxicity ^11, 17^. Administration of subtoxic doses of clofibrate (CFB), an activator of the peroxisome proliferator-activated receptor alpha (PPARα), protects mice from APAP hepatotoxicity (APAP heteroprotection) ^17^. Similarly, the APAP heteroprotection phenomenon has also been demonstrated with hepatotoxicants such as thioacetamide ^19^, CCl _4_^20, 21^, chloroform, and bromobenzene ^20^. Results from initial studies using repeated doses of CFB show that protection against APAP toxicity is associated with the prevention of GSH depletion and less covalent binding of the reactive intermediate of APAP with the cellular proteins ^17^. A subsequent study showed that a single dose of CFB prior to intoxication with APAP also protects against APAP hepatotoxicity, but neither produces changes in reactive intermediate covalent binding nor GSH depletion ^22^. Additionally, PPARα activation is a requirement for this response, since the PPARα knockout mice are not afforded protection by CFB ^23^.

In addition to APAP heteroprotection, we also demonstrated that APAP at a mildly toxic dose protects mice against hepatotoxicity from higher doses of APAP (APAP autoprotection) ^11^. Resistance to APAP-induced liver injury is also observed with repeated exposure to APAP in rats and humans ^24, 25^. Shayiq *et al.*, while describing the incremental dose model of APAP autoprotection in mice, introduced a clinical case of a physician addicted to a prescription pain medication containing a combination of an opiate and APAP ^25^. The physician claimed to have consumed 200 tablets per day in the later stages of his addiction (about 65 g of APAP) with no apparent liver damage, thus demonstrating a profound tolerance to APAP. In a later study, healthy adults given 4 g APAP per day over a period of 14 days showed elevations in plasma alanine aminotransferase (ALT) activity around days 7 to 8 that subsequently decreased towards day 14 ^26^. These studies clearly show that adaptation to hepatocyte injury occurs during repeated exposure to APAP.

Although the mechanism(s) underlying APAP autoprotection/heteroprotection is not clearly understood, a common feature in most cases is compensatory hepatocellular proliferation and resiliency of proliferating hepatocytes to further toxicant exposure. Interruption of hepatocyte replication by the antimitotic chemical colchicine restores the sensitivity of pretreated rodents to challenge with higher toxicant doses ^11, 19, 25^. Compensatory changes in biotransformation pathways (decreased bioactivation due to down-regulation of cytochrome P450 [CYP] enzymes) and enhanced conjugative capacity and/or GSH content have been proposed as potential mechanisms to explain the resiliency of the liver in these models of autoprotection and heteroprotection. However, we have shown conclusively that a single-dose APAP pretreatment alone does not alter either bioactivation (decreased CYP1A2, CYP2E1, and CYP3A11) or detoxification pathways (increased cellular GSH or UDP-glucuronosyltransferase [UGT]) ^11^. Although we showed that pretreatment alone does not alter cellular GSH levels or decrease CYP enzymes that metabolize APAP, a recent study by Eakins *et al.* showed that in APAP autoprotected livers, there is higher GSH-mediated detoxification of APAP and changes in localization of one of the CYP isoenzymes that metabolizes APAP to its toxic intermediate. They noted periportal expression of CYP2E1 in autoprotected livers as opposed to its normal centrilobular expression ^14^. The functional consequence of this change in enzyme localization is not currently known. However, CYP2E1 is a bona fide target of β-catenin, which is critical for liver zonation ^27^ and is also involved in liver regeneration after APAP overdose ^9, 10^. It is intriguing and worth noting that a similar shift in localization of flavin-containing monooxygenase-3 (FMO3) was also noted in livers of our APAP autoprotection model ^13^. Fmo3 was identified from a microarray study as a potential gene contributing to resistance to liver toxicity in our APAP autoprotection mouse model ^12^. Similar to CYP450s, FMO3 is a phase 1 drug-metabolizing enzyme involved in the oxygenation of nitrogen- and sulfur-containing endogenous substrates (such as cysteamine, trimethylamine, etc.) or xenobiotics (such as methimazole, ketoconazole, nicotine, etc.). This enzyme is not known to metabolize APAP or its metabolites. FMO3 is normally expressed in the periportal region of the female mouse liver and is nearly absent in males ^13^. However, in the APAP autoprotection model, FMO3 expression is evident in centrilobular regions of the male mouse liver, where hepatocellular damage following APAP treatment and regeneration occurs. Another gene investigated by our group in association with the APAP autoprotection model is the multidrug resistance-associated protein 4 (Mrp4), also known as ATP-binding cassette subfamily C member 4 (ABCC4). An increase in the expression of this sinusoidal efflux transporter is also seen in hepatocytes localized to centrilobular areas where compensatory hepatocellular proliferation following pretreatment with mildly toxic doses of APAP is confined ^11^. We have determined that the inclusion of the antimitotic agent colchicine in the APAP autoprotection treatment regimen not only restores the susceptibility of mice to APAP toxicity but also blunts the Mrp4 induction by APAP. Even though we have generated data supporting the role of Mrp4 as a genetic determinant of APAP hepatotoxicity, at the present time we know only that this transporter mediates the basolateral efflux of APAP-sulfate conjugates. This process by itself does not explain cellular regeneration and/or protection seen in the autoprotection mouse model. Unpublished data from our laboratory show that both male and female mice deficient in Mrp4 expression are more susceptible to hepatotoxicity by a single dose of APAP. It is plausible that Mrp4 is transporting endogenously generated molecules that, upon their efflux from hepatocytes in areas of necrosis, may have paracrine functions that strengthen the defenses of surrounding tissue and/or promote compensatory hepatocellular proliferation of neighboring cells. Alternatively, Mrp4 could be mediating reduction of intracellular concentrations of endogenous molecules normally promoting hepatocyte quiescence.

## Gene expression and proteome profiling in autoprotection/heteroprotection models

With the advent of sophisticated techniques that allow comprehensive, global evaluation of differential gene and protein expression, we have adopted gene array analysis approaches to address mechanistic toxicology questions in both heteroprotection and autoprotection models ^12, 15^. More recently, Eakins *et al.* also addressed this subject through proteomic analysis in another rodent model of APAP autoprotection involving repeated APAP administration every 24 hours for up to 96 hours ^14^. The gene array analysis performed by our group in our APAP autoprotection model identified many differentially expressed genes that could contribute to the development of resistance to APAP hepatotoxicity upon re-exposure to this toxicant. Further analysis of differentially expressed genes using a computational platform known as Causal Reasoning Engine (CRE) provided insights into candidate signaling pathways mediating autoprotection ^12^. CRE works by interrogating prior biological knowledge on the observed gene expression profile and provides hypotheses on the upstream molecular events that best explain the expression changes observed in the dataset ^28^. Induction of FMO3 was demonstrated to be prominent in APAP autoprotection. Gene and protein expression of FMO3 by APAP was thoroughly characterized, and the functional consequences of such changes in this phase I enzyme in APAP toxicity was also described ^13^. As expected, there are overlapping results from our gene expression and the proteome analysis studies by Eakins *et al.*, one of them being induction of the oxidative stress responsive transcription factor nuclear factor (erythroid-derived 2)-like 2 (Nrf2). We have shown that induction of Mrp4 during APAP hepatotoxicity is dependent on Nrf2 ^29^. On the contrary, the induction of FMO3 is not regulated by Nrf2 ^30^. Furthermore, deletion of Nrf2 alone does not abolish APAP autoprotection, since Nrf2-null mice develop tolerance to APAP hepatotoxicity similar to wild-type mice ( 14 and unpublished data from our laboratory). This is a critical observation, since two independent studies suggest that Nrf2 response likely contributes to APAP autoprotection, yet a gene loss-of-function approach demonstrated that Nrf2 response is not a prerequisite for APAP autoprotection. This also confirms the complexity and multifactorial nature of this adaptation to APAP toxicity.

Even though autoprotection is a complex phenomenon, understanding the role of individual genes that influence susceptibility to APAP toxicity is key to the development of new therapeutic strategies for ALF. To further understand the role of FMO3 in APAP toxicity, we recently developed a HepaRG™ cell line that overexpresses human FMO3. This allowed us to study the function of FMO3 in the absence of all other changes in gene expression produced by the pretreatment dose of APAP in an intact animal. HepRG cells require a special differentiation media to gain their well-documented phenotype that more closely resembles primary human hepatocytes than other hepatic cell lines available. Not only does overexpression of FMO3 alter the susceptibility of HepaRG cells to APAP toxicity but, surprisingly, it also promotes faster and more robust differentiation of this cell line in the absence of the normally required differentiation media (unpublished data from our lab). This observation is in agreement with a recent finding by Leiser *et al.*, who demonstrated that FMO enzymes increase the lifespan and enhance proteostasis in nematodes undergoing a hypoxic response, which is another form of stress/cytotoxicity ^31^. It is intriguing that FMO3, once considered to be non-inducible by xenobiotic treatment, has been shown not only to be inducible by our group and others ^12, 13, 32, 33^ but also to protect against APAP-induced cytotoxicity in hepatocytes ^13^. This highlights the evolving nature of our understanding about FMO3 function, which calls for more mechanistic studies to delineate FMO3’s role in protecting against APAP hepatotoxicity.

Currently, proposed markers for drug-induced liver injury include microRNAs (miRNAs) as liver injury markers, mechanistic biomarkers, and metabolites in urine and serum ^34^. Recent advances demonstrate the use of miRNAs as sensitive diagnostic and prognostic biomarkers for liver injury ^35^, but less progress has been made in exploiting therapeutically novel genes and pathways or circulating metabolites with contributing roles in preventing/mitigating acute liver injury. Thus, identifying key signaling molecules or catalytic products of FMO3 mediating cellular differentiation has much therapeutic usefulness. Using a targeted metabolomics approach, trimethylamine N-oxide (TMAO), a metabolite of FMO3, was identified as a factor contributing to a pro-atherogenic macrophage phenotype and cardiovascular disease ^36^. A similar approach might prove useful in cases of APAP-induced hepatotoxicity. First, circulating metabolites of FMO3 can be used as diagnostic/prognostic markers during APAP toxicity, which could help physicians to decide on the course of treatment. Alternatively, efforts can be focused to stabilize and/or increase FMO3 in hepatocytes during APAP toxicity that could help drive hepatocytes toward regeneration. Another alternative is the exogenous administration of a yet-to-be-determined FMO3 catalytic product with hepatoprotective or tissue regenerative properties.

Gene microarray analysis in an APAP heteroprotection model from our laboratory identified a significant increase in the expression of the enzyme Vanin-1 (Vnn1), whose basal expression and induction by CFB are dependent on the presence of PPARα ^15^. The *Vnn1* gene encodes for a member of the Vanin family of proteins that possess pantetheinase activity. Pantetheinase hydrolyzes pantetheine into pantothenic acid (vitamin B5) and cysteamine and may also play a role in the oxidative stress response. We recently documented the role of Vnn1 as a novel genetic determinant of APAP toxicity. Vnn1’s role during APAP toxicity was confirmed using Vanin-1 knockout mice. These mutants have heightened sensitivity to APAP-induced hepatotoxicity, which we attributed to a deficiency in compensatory hepatocellular proliferation and deficient infiltration of immune cells into the liver following APAP treatment. Vnn1 expression confers protection to APAP and also to CCl _4_ and concanavalin A ^16^. It is intriguing that the increased Vnn1 expression after CFB treatment in the APAP heteroprotection CFB/PPARα model and the increased FMO3 expression in the APAP autoprotection model both play an upstream and downstream role in cysteamine metabolism, respectively. While Vnn1 plays a role in the synthesis of cysteamine, FMO3 mediates the further metabolic N-oxygenation of cysteamine. Cysteamine is involved in several critical pathways of cellular homeostasis including lipid catabolism, synthesis of cholesterol, taurine, and nicotinamide adenine dinucleotide plus hydrogen (NADH), as well as maintenance of cellular redox balance. Although the exact roles of Vnn1, FMO3, and cysteamine metabolism in APAP heteroprotection/autoprotection have not been clearly defined, the over-expression of both Vnn1 and FMO3 is protective against APAP-induced hepatotoxicity. Additional studies should explore the functional relationship of these two genes as determinants of hepatotoxicant susceptibility.

## Summary

Understanding the mechanisms of xenobiotic-induced liver injury and recovery, which include liver tissue regeneration, remains a challenge. Considerable progress has been made to delineate the mechanisms of injury. In spite of this, liver damage is still largely assessed based on ALT enzyme levels in the blood and the only FDA-approved treatment available is NAC to replenish cellular GSH levels. Although the role of circulating metabolites of enzymes that are differentially expressed in these models of APAP tolerance development and their potential use in diagnosis and/or prognosis is now well appreciated, the only product that has reached clinical testing is the adduct dipstick for the diagnosis of APAP toxicity (ClinicalTrials.gov identifier: NCT01575847). The discovery of protein dynamics during autoprotection using proteomics has shed light onto the complexity and plasticity of hepatocytes during stress conditions. The identification of Vnn1 as a genetic determinant of APAP hepatotoxicity has revealed the existence of an immune regulator that could help elucidate which population of immune cells migrate to the injured liver and their specific role in dictating the course and ultimate outcome of toxicant challenge. Finally, the realization that FMO3 might play an important role in cellular differentiation (unpublished observation by our laboratory) has provided new and exciting insights into the potential function(s) of FMO3 beyond its well-characterized functions as a drug-metabolizing enzyme. Future studies will be instrumental to identifying circulating metabolites (that are common between autoprotected rodent livers and human livers recovering from injury), like those generated by FMO3, to use as prognostic tools in APAP-overdose patients. Despite the progress we have discussed, much more work is needed to develop novel therapeutic interventions for acute liver damage. Fortunately, based on the research presented here, a promising future avenue for the development of compounds to treat acute liver damage is to target the stabilization of proteins contributing to tissue remodeling and/or the differentiation of proliferating hepatocytes.

## Abbreviations

APAP, acetaminophen; ALF, acute liver failure; CYP, cytochrome P450; FMO3, flavin-containing monooxygenase-3; Mrp4, multidrug resistance-associated protein-4; NAC, N-acetyl cysteine; NADH, nicotinamide adenine dinucleotide plus hydrogen; Nrf2, nuclear factor (erythroid-derived 2)-like 2; OLT, orthotopic liver transplantation; PPARα, peroxisome proliferator-activated receptor alpha.
